# Natural Products and Nutrients against Different Viral Diseases: Prospects in Prevention and Treatment of SARS-CoV-2

**DOI:** 10.3390/medicina57020169

**Published:** 2021-02-14

**Authors:** Syed Ghazanfar Ali, Mohammad Azam Ansari, Mohammad A. Alzohairy, Ahmad Almatroudi, Mohammad N. Alomary, Saad Alghamdi, Suriya Rehman, Haris M. Khan

**Affiliations:** 1 Viral Research Diagnostic Laboratory, Department of Microbiology, Jawaharlal Nehru Medical College A.M.U., Aligarh U.P.202002, India; harismk2003@hotmail.com; 2 Department of Epidemic Disease Research, Institutes for Research and Medical Consultations (IRMC), Imam Abdulrahman Bin Faisal University, Dammam 31441, Saudi Arabia; maansari@iau.edu.sa (M.A.A.); surrehman@iau.edu.sa (S.R.); 3 Department of Medical Laboratories, College of Applied Medical Sciences, Qassim University, Qassim 51431, Saudi Arabia; dr.alzohairy@gmail.com (M.A.A.); aamtrody@qu.edu.sa (A.A.); 4 National Centre for Biotechnology, King Abdulaziz City for Science and Technology (KACST), P.O. Box 6086, Riyadh 11442, Saudi Arabia; 5 Laboratory Medicine Department, Faculty of Applied Medical Sciences, Umm Al-Qura University, Makkah21955, Saudi Arabia; ssalghamdi@uqu.edu.sa

**Keywords:** antiviral, medicinal plants, pandemic, SARS-CoV-2, zoonotic virus

## Abstract

Severe acute respiratory syndrome coronavirus-2 (SARS-CoV-2) has caused a global pandemic and is posing a serious challenge to mankind. As per the current scenario, there is an urgent need for antiviral that could act as a protective and therapeutic against SARS-CoV-2. Previous studies have shown that SARS-CoV-2 is much similar to the SARS-CoV bat that occurred in 2002-03. Since it is a zoonotic virus, the exact source is still unknown, but it is believed bats may be the primary reservoir of SARS-CoV-2 through which it has been transferred to humans. In this review, we have tried to summarize some of the approaches that could be effective against SARS-CoV-2. Firstly, plants or plant-based products have been effective against different viral diseases, and secondly, plants or plant-based natural products have the minimum adverse effect. We have also highlighted a few vitamins and minerals that could be beneficial against SARS-CoV-2.

## 1. Introduction

Coronaviruses under the realm of Riboviria belongs to the order Nidovirales, suborder cornidovirinea, family Coronoviridae [1]. Orthocoronavirinae the subfamily of coronoviridae is further divided into four genera alpha (a), beta (b), gamma (c), and delta (d) coronavirus. Further, SARS-CoV belongs to Beta coronavirus and sarbecovirus is the subgenus of SARS-CoV-2 [2]. It shares similar homology with bat coronavirus and it has been estimated that both have 96.2% sequence homology [3].

The two epidemics due to coronavirus have already occurred that were due to SARS-CoV and the Middle East respiratory syndrome (MERS) and both have been the major cause of pneumonia in humans [4]. SARS-CoV emerged in 2002 and remained till 2003 during the period it caused 774 death and more than 800 people were infected. MERS-CoV emerged in Saudi Arabia in 2012, which caused more than 800 deaths, and 2500 people became infected [5].

Coronaviruses have single-stranded RNA as genetic material that can range from 26 kbs to 32 kbs in length. Coronaviruses possess specific genes in Open Reading Frame ORF1 downstream region that are responsible for encoding the proteins responsible for viral replication, nucleocapsid, and spike formation [6]. The structure of SARS-CoV shows the spikes on the outer surface. Furthermore, studies have shown that structural proteins are encoded by the four structural genes, which include spike (S), envelope (E), membrane (M), and nucleocapsid (N) gene. These genes are responsible for viral functionality and structure [7].

SARS-CoV-2 genome is 80% similar to the previous human coronavirus SARS-CoV [8]. For some of the encoded proteins such as coronavirus main proteinase (3CLpro), papain-like protease (PLpro), and RNA-dependent RNA polymerase (RdRp) the sequence similarity is very high (96%) between SARS-CoV, and SARS-CoV-2 [9].

SARS-CoV-2 and SARS-CoV spike protein have 76.5% identical amino acid sequences [10]. The 3D structures of spike proteins of SARS-CoV-2 and SARS-CoV as analyzed by computer imaging revealed that both have identical structures in the receptor-binding domain and maintains the Vander Waals force [10]. The major amino acid residue of SARS-CoV-2 in the receptor-binding domain is Gln-493, which favors the attachment of spike protein S with human cells more specifically with the lungs therefore the attachment results in respiratory infections in humans [11,12]. Zhao et al. [11] have suggested that 83% of the angiotensin-converting enzyme (ACE-2) expressing cells are alveolar epithelial type II, therefore, these cells can be the reservoir for the viral invasion. Some analysts have suggested that SARS-CoV-2 binds to ACE-2 more efficiently as compared with SARS-CoV, therefore, it has a greater ability to transmit from person to person [13].

Initially, for the attachment to the host cells, virus use the spike (S) glycoprotein, which also mediates the host and viral cell membrane fusion during infection [14]. The spike (S) glycoprotein has two regions namely S1 and S2. S1 helps in the binding of host cell receptors, and S2 helps in the fusion with the membrane. The receptor-binding domain (RBD) is located in the S1 region of SARS-CoV and the attachment of the human host cell with the virus is mediated by the RBD protein of the RBD domain with the angiotensin-converting enzyme (ACE-2) as a receptor similar to the one required by SARS-CoV [15,16]. Since SARS-CoV and SARS-CoV-2 are much similar, it is believed that SARS-CoV-2 uses a similar receptor (ACE-2) for entering into human host cells [10,13].

The initial clinical symptoms that appear after SARS-CoV-2 infection are pneumonia, fever, dry cough, headache dyspnea, and in acute cases leads to respiratory failure and eventually, death occurs [17,18,19]. Although the healing depends upon immunity, pre-existing conditions such as hypertension, diabetes, cardiovascular diseases, or kidney diseases further enhances the severity of the pathogenesis of SARS-CoV-2 [17,19]. Different drugs are available in the market against SARS-CoV-2 but none has shown accurate results. None of the drugs has been approved by FDA against SARS-CoV-2.

Plants have been used as a medicine in the traditional Chinese and Ayurvedic systems [20]. Some of the herbal medicines have safety margins above those of reference drugs, which shows that they can be used against mild SARS-CoV-2 infection [21]. Plants in the form of herbal medicine or dietary components act as immunomodulators and can be a potent antiviral against SARS-CoV-2 infection [22].

Medicinal plants contain diverse secondary metabolites, therefore, they have been the source of drugs against viral, bacterial, and protozoal infection, including cancer [23,24]. The secondary metabolites present in the medicinal plants can interrupt viral proteins and enzymes by binding with them and preventing viral penetration and replication [25,26,27,28]. When consumed without knowing the appropriate dosage, herbal medicines may be toxic [29]. The genetically modified (GM) plants also pose serious health issues because the unnatural change in naturally occurring protein or the metabolic pathways results in toxins or allergens [30]. Therefore, the selection of medicinal plants is also an important aspect.

Vitamins play a major role in preventing established respiratory infection and intensive care settings. Vitamins enhance natural barriers because of their antioxidant and immunomodulatory effect and this could be beneficial against SARS-CoV-2 [31]. Mineral elements such as selenium and zinc have shown antiviral effects by enhancing the immune responses [32,33].

In this review article, we have tried to highlight the use of medicinal plants against different viral diseases including SARS-CoV-2, and also the possible factors (vitamins and minerals) that could be helpful against SARS-CoV-2.

## 2. Plants as a Source of Medicine against SARS-CoV-2

### 2.1. Glycyrrhiza Glabra

*Glycyrrhiza glabra* belongs to the family Fabaceae and is commonly known as licorice. Dried roots from the plant have a characteristic odor and sweet taste. Glycyrrhizin is the main component isolated from the roots. Glycyrrhizin inhibited 50% hepatitis C virus (HCV) in a dose-dependent manner at a concentration of 14 ± 2 µg [34].

Glycyrrhizin also protects from the influenza A virus and caused a reduction in influenza-infected lung cells [35]. In addition to being effective against hepatitis and influenza virus, it has also shown its effect on SARS CoV [31]. Cinatal et al. [36] showed that glycyrrhizin effectively inhibited the SARS-CoV virus replication at an early stage. They were also of opinion that glycyrrhizin affected cellular signaling pathways such as protein kinase C, casein kinase II, and transcription factors such as protein 1 and nuclear factor B. Furthermore, they also concluded the upregulation of nitrous oxide that inhibits the virus.

Sinha et al. [37] through in silico approach showed that the replication process of SARS-CoV-2 was affected by the two components of licorice (glyasperin A and glycyrrhizic acid) They concluded that the ACE-2 receptor of the virus was disturbed by glycyrrhizic acid and the replication process was inhibited by glyasperin A (Table 1).

### 2.2. Alnus Japonica

*Alnus japonica*, also known as the East Asian alder tree, grows approximately up to 22 m. It is deciduous with a very fast growth rate. It belongs to the family Betulaceae, also known as the birch family, which includes six genera having nut-bearing deciduous trees and shrubs. The extract of leaves and bark of *Alnus japonica* have been used as food that boosts immunity against influenza [38]. Studies have shown that methanolic extract of *Alnus japonica* bark possesses strong antiviral activity against the H9N2 subtype avian influenza virus [39]. Further, the chromatographic separation revealed the presence of four lupine type triterpenes and one steroid [39].

Diarylheptanoids isolated from *Alnus japonica* showed inhibitory activity against papain-like protease (required for replication of SARS-CoV) [40]. Nine diarylheptanoids were isolated, namely, platyphyllenone, hirsutenone, hirsutanonol, oregonin, rubranol, rubranoside B, and rubranoside A. Furthermore, hirsutenone, hirsutanonol, oregonin, rubranol, rubranoside B, and rubranoside A showed dose-dependent inhibitory activity against the papain-like protease. They also showed that hirsutenone possessed the most potent inhibitory activity against papain-like protease. Furthermore, the analysis of Park et al. [40] showed that catechol and α,β-unsaturated carbonyl moiety were present in the molecule, which are the key requirements for SARS-CoV cysteine protease inhibition.

Furthermore, Kwon et al. [41] described that *Alnus japonica* extract is useful for prevention from viral diseases, such as influenza of humans and of other mammalian and avian species. They also said that the extract exhibits very low toxicity in normal cell conditions but has high antiviral activity. Lastly, the extract from *Alnus japonica* could be effectively used in foods and pharmaceutical industries against the influenza virus.

### 2.3. Allium Sativum(Garlic)

*Allium sativum* belongs to the Amaryllidaceae family. It is a perennial flowering plant that grows from a bulb and comprises a tall flowering stem that attains a height of upto 1 m. *Allium sativum* is commonly called garlic, which is also an ingredient in Indian food. A peculiar smell can be noticed from the bulb of garlic, which is due to the presence of sulfur compounds. In addition to possessing antibacterial activity, *Allium sativum* (garlic) also has antiviral properties. *Allium sativum* extract hasan inhibitory effect on avian infectious bronchitis virus, which is a single-stranded RNA and belongs to coronaviruses. It has also shown its effect on other viruses including influenza A and B [42], cytomegalovirus [43], herpes simplex virus 1 [44], and herpes simplex virus-2 [45].

Molecular docking studies have revealed that garlic can inhibit SARS-CoV-2 [46]. Molecular docking has shown that 17 organosulfur compounds from garlic essential oil can effectively interact with the amino acid of angiotensin-converting enzyme (ACE-2), a receptor in the human body and main protease (PDB6LU7) of SARS-CoV-2. The highest efficacy was observed by allyl disulfide and allyl trisulfide. Furthermore, it was predicted that garlic essential oil or extract can be used as a source of antiviral against SARS-CoV-2 [46].

### 2.4. Houttuynia Cordata

*Houttuynia cordata* belongs to the family Saururaceae and is mostly confined to the moist habitat. It is an aromatic medicinal plant that has a creeping rootstock. Ethyl acetate extract of *Houttuynia cordata* is effective in inhibiting the infectivity of the dengue virus (DENV). Some workers have shown the inhibition of DENV-2 by ethyl acetate extract of *Houttuynia cordata* [47,48]. *Houttuynia cordata* extract also inhibited the avian infectious bronchitis virus [49]. Quercetin 3-rhamnoside from *Houttuynia cordata* showed anti-influenza (inf-A) activity and inhibited virus replication in the initial stages of infection [50]. Stem distillate from the *Houttuynia cordata* has shown inhibitory activity against herpes simplex virus-1, influenza virus, and human immunodeficiency virus-1 without any cytotoxicity [51]. The three major components from the distillate were methyl *n*–nonyl ketone, lauryl aldehyde, and capryl aldehyde.

*Houttuynia cordata* showed significant inhibition on SARS-CoV. The *Houttuynia cordata* (HC) extract successfully inhibited the SARS-CoV3C such as protease (3CLpro) and RNA-dependent RNA polymerase (RdRp) [52]. Furthermore, it was also observed that *Houttuynia cordata* extract was non-toxic to animal models at an oral administration dose of 16 g/kg. Through flow cytometric analysis, Lau et al. [52] showed that *Houttuynia cordata* extract in mice model significantly increases the CD4+ and CD8+ T cells with a significant increase in the secretion of IL-2 and IL-10 cytokine in mouse splenic lymphocytes.

### 2.5. Lycoris Radiata

*Lycoris radiata*, which is commonly known as red spider lily, hell flower, red magic lily, or sometimes called equinox flower, belongs to the family Amaryllidaceae. *Lycoris radiata* has shown its effect against H5N1 [53]. Lycorine the active component of *Lycoris radiata* successfully inhibited the influenza virus, which is responsible for avian influenza infection [54]. Proteomic analysis revealed that lycorine alters protein expression in avian influenza virus-infected cells and the treatment with lycorine also decreased the levels of nuclear pore complex protein 93(Nup93, E2RSV7), which is a part of nuclear-cytoplasmic transport [54]. Lycoricidine derivatives obtained from the bulb of *Lycoris radiata* have also shown the anti-hepatitis C virus activity [55]. Furthermore, it has also been observed that Lycorocidine derivates possess inhibitory activity against the tobacco mosaic virus [56].

Ethanolic extract of the stem cortex of the plant successfully inhibited the SARS-CoV. [57]. The active component that probably inhibited the SARS-CoV was lycorine (C16H17NO4), which was analyzed by high-performance liquid chromatography (HPLC). Although the mechanism of action of lycorine on the SARS-CoV is still unclear, it has shown promising results as an antiviral. Li et al. [57] also showed that commercially available lycorine inhibited the SARS-CoV.

### 2.6. Tinospora Cordifolia

*Tinospora cordifolia*, commonly called Guduchi or Giloy, belongs to the family Menispermaceae. The plant is an herbaceous vine that bears heart-shaped leaves. *Tinospora cordifolia* extract has shown an increase in IFN-α, IL-1, IL-2,and IL-4 levels in the peripheral blood mononuclear cells in the chickens infected with infectious bursal disease virus, which causes infectious bursal disease [58]. Further, the reduction in the mortality rate of chickens treated with *Tinospora cordifolia* extract was also observed when compared with uninfected chickens [58].

The stem extract of *Tinospora cordifolia* has antibacterial, antifungal, and antiviral properties. The stem extract of *Tinospora cordifolia* successfully inhibited the herpes simplex virus by 61.43% in the Vero cell line [59]. Kalikar et al. [60] showed that 60% of the patients who received *Tinospora cordifolia* extract and 20% on placebo showed a decrease in the incidence of various symptoms associated with HIV. However, they did not study all the parameters but hypothesized that *Tinospora cordifolia* extract could be used as an adjunct to HIV management. The in silico approach against SARS-CoV-2 was tested by Sagar and Kumar [61]. The key targets they considered for the docking were surface glycoprotein, receptor-binding domain, RNA-dependent RNA polymerase, and protease. The natural compounds from *Tinospora cordifolia* such as berberine, isocolumbin, magnoflorine, and tinocordiside showed high binding efficacy against all four key targets [61].

### 2.7. Vitex Trifolia

*Vitex trifolia* belongs to the family Verbenaceae. The leaves of the plants are compound and oppositely arranged with three linear leaflets ranges between1–12 cm in length. The upper surface of the leaf is mostly green, whereas the lower surface is grayish.

Fruits of the plants contain essential oils, monoterpenes, diterpenes, beta-sitosterol [62,63,64,65,66,67,68]. The leaves and bark of the plant contain essential oils, flavones, and artemetin [65]. Furthermore, the essential oil of *Vitex trifolia* is used as an anti-inflammatory and against headache, cold, cough, liver disorders, and HIV [66,67]. Extract from the leaves of *vitex trifolia* inhibited TNF-α and IL-1 β production in human U937 macrophages [68]. The components that were isolated from leaf extract were artemetin, casticin, vitexilactone, and maslinic acid. Wee et al. [68] also suggested that artemetin could be the active compound that suppressed TNF-α and IL-1β production.

Due to the high contents of the phenolic compounds such as phenolic acid, flavones, and flavonols, it was analyzed that methanolic extract contains antioxidant and anticancer properties [69]. *Vitex trifolia* has also been found to block the NK-kB pathways that reduce inflammatory cytokines; this is a similar pathway that is being used in respiratory distress in SARS-CoV [70,71].

## 3. Vitamins and Minerals

Vitamins and minerals are an essential requirement of our body since they help in wound healing, enhancing the immune system, etc. They are also required for converting food into energy and play a major role in cellular damage. There are two types of vitamins, fat-soluble and water-soluble, and here we have discussed only those vitamins and minerals that could have a probable role against SARS-CoV (Table 2).

Vitamin A also called retinol is the fat-soluble vitamin that has β-carotene as its plant-derived precursor. Vitamin A is also called as an anti-infective vitamin since many of the defensive mechanism of the body depends upon the supply of this vitamin. It has already been reported that calves having low vitamin A content are more susceptible to the infection since they lose the effectiveness of the bovine coronavirus vaccine [72]. Bronchitis virus, which is a kind of coronavirus, was more prominent in the chickens that were fed with vitamin A deficient diet [73].

Vitamin B is a complex of different vitamins together designated as vitamin B complex because it comprises of different vitamins (B1 and B2 up to B-12). Each vitamin has its specific requirement, and its deficiency is related to different diseases. Elderly people are more prone to vitamin B2 deficiency [74]. Vitamin B2, along with UV light, effectively reduced the titer of MERS-CoV in human plasma products [75]. Vitamin B3 inhibited neutrophil infiltration into the lungs causing a strong anti-inflammatory response during the ventilator-associated lung injury but also led to hypoxemia [76]. Vitamin B-12 improves sustained viral response in patients with hepatitis C [77]. Narayan and Nair [78], through computational analysis using different software, also predicted that Vitamin B12 inhibited RNA-dependent RNA polymerase activity of an nsp-12 protein in SARS-CoV-2. nsp-12protein is associated with RNA-dependent RNA polymerase activity and is responsible for viral replication.

Vitamin C has shown a significant lowering of pneumonia in lower respiratory tract infections during clinical trials [79]. Therefore, it is suggested that vitamin C might work against SARS-CoV-2. There are reports that suggest that vitamin C had increased the resistance of chicken embryo organ culture against avian coronavirus infection [80].

Vitamin D is a prohormone vitamin and is produced during exposure to sunlight, although smaller amounts are obtained from the food as well. It has many roles, including the enhancement of cellular immunity through the induction of antimicrobial peptides [81,82]. SARS-CoV-2 infection can be lowered by targeting an unbalanced Renin-angiotensin system (RAS) with Vitamin D supplements [83]. The current pandemic of SARS-CoV-2 has led to a call for the widespread use of vitamin D supplements [83,84,85,86]. Some reviews have suggested vitamin D loading doses of 200,000–300,000 IU in 50,000 IU capsules reduce the severity of COVID-19 [87].

Vitamin E, along with Vitamin D, deficiency has caused the bovine coronavirus in calves [88].

Zinc is required for the functioning and maintenance of immune cells. Zinc also increases interferon α (IFNα) production by leukocytes [89] and mediates its antiviral activity in cells infected with the rhinovirus [90]. In combination with pyrithione, zinc inhibits the RNA transcription in SARS-CoV and SARS-CoV RNA polymerase activity in RNA-dependent RNA polymerase. Selenium could also be another alternative to treat against SARS-CoV. The synergistic effect of selenium with ginseng stem-leaf saponins could induce an immune response to a bronchitis coronavirus vaccine in chicken [91].

## 4. Conclusions

From this review, we attempted to highlight natural medicines that could be used against SARS-CoV-2. Different companies and institutes have launched vaccines after getting approval from World Health Organization (WHO), whereas some vaccines are still under clinical trials. Different antivirals that have been previously used against SARS, MERS, and influenza have been evaluated alone or in combination, but still, no fruitful response has been achieved. Furthermore, it has been reported that the virus has a high rate of mutation, which is again a hurdle in the development of a vaccine [92].

This review is based on the treatments given in the case of SARS-CoV and MERS, which could also be effective against SARS-CoV-2 since SARS-CoV-2 is closely related to SARS-CoV and have 80% sequence identical [8]. Plants are a good source of natural medication and an alternative to antibiotic treatment because the excessive use of antibiotics is responsible for drug resistance. Secondly, the main advantage of using plants is that they are economically efficient, have high scalability and safety because the plants can be cultivated in a very low amount, and they also do not support the growth of human pathogens [93]. Plants have produced a large range of antivirals lectins, including griffithsin [94,95], cyanovirin [96,97,98,99] and cyanovirin-N-fusion proteins [100], and also the transgenic rice lines that express griffithsin and cyanovirin-N in the seeds, with or without antibody 2G12 [101]. Medicinal plants may be a good source of antivirals, but the accurate dosage and plant parts that have medicinal properties, such as root, shoot, etc., should be known prior to the consumption; otherwise, the adverse effect could also exist. Although the exact mechanism of action of plants on to the virus is still unknown, we are of opinion that healthy food helps in boosting the immune system, which further prevents disease including SARS-CoV-2. Therefore, a nutritious diet that includes different vitamins, minerals should be consumed regularly.

## 5. Future Perspectives

Plants could be a better alternative to modern medicines in the future. To validate the hypothesis of plants as a medicine further, extensive research should be carried out on different medicinal plants so that correct dosage and toxicity levels may be known prior to application.

## Figures and Tables

**Table 1:** Plants and their activity against different viral diseases.

Plants	Family	Plant Part	References
*Glycyrrhiza glabra*	Fabaceae	Roots	Hepatitis C [34] Influenza-A [35] SARS-CoV [36] SARS-CoV-2 [37]
*Alnus japonica*	Betulaceae	Leaves and bark	Influenza [38] H9N2 Avian influenza virus [39] SARS CoV [40]
*Allium sativum*	Amaryllidaceae	Bulb	Influenza A and B [42] Cytomegalovirus [43] Herpes simplex virus 1 [44] Herpes simplex virus 2 [45] SARS CoV [46]
*Houttuynia cordata*	Saururaceae	Leaves, roots	DEN V-2 [47,48] Avian infection bronchitis virus [49] Influenza-A [50] Herpes simplex virus-1 [51] SARS CoV [52]
*Lycoris radiata*	Amaryllidaceae	Bulb, stem cortex	H5N1 [53] Hepatitis C [55] Tobacco mosaic virus [56] SARS CoV [57]
*Tinospora cordifolia*	Menispermaceae	Stem	HSV-1 [59] HIV [60] SARS-CoV [61]
*Vitex trifolia*	Verbenaceae	Leaves	HIV [66,67] SARS-CoV [70,71]

**Table 2:** Vitamins and their role.

Vitamins	Role
Vitamin-A	Deficiency of Vit-A leads to enhancement of Bronchitis virus in chicken [73]
Vitamin B2	Effectively reduced titer of MERS-CoV in human plasma products [75]
Vitamin B3	Strong anti-inflammatory response during the ventilator-associated lung injury [76]
Vitamin B-12	Improves sustained viral response in patients with hepatitis C [77] Inhibits RNA Dependent RNA polymerase activity of an nsp-12 protein in SARS-CoV-2 [78]
Vitamin C	Lowering of pneumonia in lower respiratory tract infection [79]. Increase the resistance of chicken embryo organ culture against avian coronavirus infection [80]
Vitamin D	Enhancement of cellular immunity through induction of antimicrobial peptides [81,82]. Lowering of SARS-CoV-2 infection by targeting unbalanced RAS [83].
Vitamin E	Deficiency of Vit-E along with Vit-D caused the bovine coronavirus in calves [88]

## Data Availability

Not Applicable.
